# The Potential of Sulfated Polysaccharides Isolated from the Brown Seaweed *Ecklonia maxima* in Cosmetics: Antioxidant, Anti-melanogenesis, and Photoprotective Activities

**DOI:** 10.3390/antiox9080724

**Published:** 2020-08-09

**Authors:** Lei Wang, Thilina U. Jayawardena, Hye-Won Yang, Hyo-Geun Lee, You-Jin Jeon

**Affiliations:** 1 Department of Marine Life Sciences, Jeju National University, Jeju Self-Governing Province, Jeju 63243, Korea; comeonleiwang@163.com (L.W.); tuduwaka@gmail.com (T.U.J.); koty221@naver.com (H.-W.Y.); hond0502@hanmail.net (H.-G.L.); 2 Marine Science Institute, Jeju National University, Jeju Self-Governing Province, Jeju 63243, Korea

**Keywords:** *Ecklonia maxima*, sulfated polysaccharides, oxidative stress, melanogenesis, UVB irradiation

## Abstract

Sulfated polysaccharides prepared from marine algae are potential ingredients in nutraceutical, pharmaceutical, and cosmeceutical industries. In the present study, the antioxidant, anti-melanogenesis, and photoprotective effects of sulfated polysaccharides obtained from *Ecklonia maxima* (EMC) were investigated to evaluate their potential in cosmetic. EMC was successfully prepared through Celluclast-assisted extraction and ethanol precipitation, and it contained 79.88% of sulfated polysaccharides that with 69.37% carbohydrates and 10.51% sulfate. EMC effectively suppressed 2,2-azobis(2-amidinopropane) hydrochloride (AAPH)-induced oxidative stress in vitro in Vero cells and in vivo in zebrafish. Furthermore, EMC significantly inhibited mushroom tyrosinase and reduced melanin synthesis in alpha-melanocyte-stimulating hormone-stimulated B16F10 cells. In addition, EMC remarkably attenuated photodamage induced by UVB irradiation in vitro in human keratinocytes (HaCaT cells) and in vivo in zebrafish. Furthermore, EMC effectively inhibited wrinkle-related enzymes and improved collagen synthesis in UVB-irradiated human dermal fibroblasts (HDF cells). These results indicate that EMC possesses strong antioxidant, anti-melanogenesis, and photoprotective activities, and suggest that EMC may be an ideal ingredient in the pharmaceutical and cosmeceutical industries.

## 1. Introduction

Skin is the largest organ of the human body and plays various physiological functions. The skin is directly exposed to the environment. Thus, it is continually damaged by environmental factors such as chemicals, fine dust particles, and ultraviolet (UV) irradiation [1]. These environmental factors cause various effects on the skin including oxidative stress, inflammatory responses, melanin accumulation, and collagen degradation, leading to skin aging [2,3,4,5]. The appearance of the skin directly contributes to personal beauty [6]. Some reports indicated that people are more satisfied with their life if their skin looks younger than their actual age [6,7]. Thus, a skincare product which could remarkably suppress skin aging may have a great market demand. In recent decades, the discovery of an ingredient from natural resources to be used in cosmeceutical industries has attracted researcher’s attention. 

Seaweeds are rich in natural compounds such as fatty acids, phenolic compounds, sterols, proteins, and polysaccharides [8,9,10,11,12]. In particular, seaweeds possess a high amount of polysaccharides [13]. Sulfated polysaccharides isolated from seaweeds have been reported to produce numerous health benefits such as activating the immune system, antioxidant, anti-coagulant, UV protective, liver protective, anti-inflammatory, antimicrobial, and anti-cancer activities [14,15,16,17,18,19,20,21]. Wang et al. (2019) isolated sulfate polysaccharides (HFPS) from the brown seaweed *Hizikia fusiforme* and evaluated the UV protective effects of HFPS [15]. The results indicated that HFPS effective protects against UVB-induced photodamage in both in vitro and in vivo models [15]. Marques et al. (2019) isolated sulfated polysaccharides (UFPS) from the greed seaweeds *Udotea flabellum* and evaluated anticoagulant and antitumor activities of UFPS [19]. The results indicated that UFPS possesses strong anticoagulant and antitumor effects [19]. 

*Ecklonia maxima* is a brown seaweed typically found along the southern Atlantic coast of Africa. Polysaccharides from *E. maxima* have been reported to have anti-diabetes, anticancer, and antioxidant activities [22,23,24]. However, the cosmeceutical potential of polysaccharides isolated from *E. maxima* has not been evaluated so far. The objectives of the present research are as follow: to isolate the polysaccharides from the brown seaweed *E. maxima* through enzyme-assisted extraction; to evaluate the potential of the polysaccharides from *E. maxima* in the cosmeceutical industry. In the present study, the polysaccharides from *E. maxima* were prepared by enzyme-assisted extraction, and their cosmeceutical potential were evaluated through evaluating their antioxidant, anti-melanogenesis, and UV protective effects. 

## 2. Materials and Methods

### 2.1. Chemicals and Regents

Dulbecco’s modified Eagle medium (DMEM), Roswell Park Memorial Institute-1640 (RPMI-1640) medium, Ham’s nutrient mixtures medium (F-12 medium), penicillin-streptomycin (P/S), trypsin-EDTA, and fetal bovine serum (FBS) were purchased from Gibco-BRL (Grand Island, NY, USA). PIP ELISA kit was purchased from TaKaRa Bio Inc (Shiga, Japan). The 3-(4,5-Dimethylthiazol-2-yl)-2,5-diphenyltetrazolium bromide (MTT), α-melanocyte-stimulating hormone (α-MSH), acridine orange, 2,2-azobis(2-amidinopropane) hydrochloride (AAPH), 1,3-Bis (diphenylphosphino) propane (DPPP), 2,7-dichlorofluorescein diacetate (DCFH2-DA), diaminofluorophore 4-amino-5-methylamino-2′,7′-difluorofluorescein diacetate (DAF-FM-DA), (Kusatsu, Japan) and the ELISA kits used for the analysis of human matrix metalloproteinases (MMPs) were purchased from Sigma (St. Louis, MO, USA). All other chemicals used in this study were of analytical grade.

### 2.2. Determination of the Approximate Composition of E. maxima 

The seaweed *E. maxima* was kindly provided by Prof. John J. Bolton, University of Cape Town, South Africa. The approximate composition of *E. maxima* including moisture, ash, protein, lipid, and carbohydrate contents, was determined according to the methods described by the Association of Official Analytical Collaboration (AOAC) International [25].

### 2.3. Preparation of Polysaccharides from E. maxima 

The lyophilized seaweed powder (10 g) was hydrolyzed with Celluclast from *Trichoderma reesei* (Sigma, St. Louis, MO, USA, ≥700 units/g) (1:100:5, *w/v/v*) for 24 h under the optimal conditions (pH 4.5, 50 °C). After hydrolysis, the enzyme was inactived (100 °C, 10 min) and the pH was adjusted to 7 with 1 M NaOH. Then the Celluclast-assisted extract of *E. maxima* was obtained and named EM. EM was mixed with 95% ethanol (1:2, *v/v*) and kept at 4 °C for 24 h. Then, the precipitates were collected and considered as the crude polysaccharides of *E. maxima*, which are referred as EMC.

### 2.4. FT-IR Characterization

The FT-IR spectra of EMC and the commercial fucoidan were analyzed using an FT-IR spectrometer (Bruker, Karlsruhe, Germany) according to a previously described protocol [26].

### 2.5. Evaluation of the Enzyme Inhibitory Effect of EMC

The inhibitory effect of EMC on tyrosinase was determined according to the protocol described in at previous study [27]. In addition, the inhibitory effects of EMC on elastase and collagenase were determined according to the methods described by Wang et al. [28].

### 2.6. Maintenance of Cell Lines and Zebrafish

Monkey kidney fibroblasts (Vero cells, ATCC^®^CCL-81™, Manassas, VA, USA) were cultured in RPMI-1640 medium (10% FBS and 1% P/S) and seeded at a density of 1 × 10^5^ cells/mL for experiments. Human epidermal keratinocytes (HaCaT cells, ATCC^®^ PCS-200-001™, Manassas, VA, USA) and mouse melanoma cells (B16F10 cells, ATCC^®^CRL-6475™, Manassas, VA, USA) were cultured in DMEM medium (10% FBS and 1% P/S). HaCaT cells and B16F10 cells were seeded at a density of 1 × 10^5^ and 3 × 10^4^ cells/mL, respectively. Human dermal fibroblasts (HDF cells, ATCC^®^PCS201012™, Manassas, VA, USA) were cultured in the medium mixed with F-12 and DMEM (1:3) supplemented with 10% FBS and 1% P/S. HDF cells were seeded at a concentration of 5.0 × 10^4^ cells/mL for experiments.

The zebrafish were maintained following the conditions described previously [14,15]. The experiment was approved by the Animal Care and Use Committee of the Jeju National University (Approval No. 2019-O-0074).

### 2.7. Determination of the Effect of EMC on AAPH-Induced Oxidative Stress 

#### 2.7.1. In Vitro Assay

To investigate the in vitro antioxidant activity of EMC, the protective effect of EMC against AAPH-induced oxidative stress was determined. Vero cells were seeded in a 24-well plate and incubated for 24 h. The cells were treated with EMC and stimulated with AAPH. The intracellular ROS scavenging and cytoprotective effects of EMC on AAPH-stimulated Vero cells were evaluated by the DCF-DA and the MTT assays, respectively [29,30]. In addition, the effect of EMC on AAPH-induced apoptosis was determined with the Hoechst staining assay according to the protocol described by Wang et al. [31].

#### 2.7.2. In Vivo Assay

The in vivo antioxidant activity of EMC was investigated in zebrafish stimulated with AAPH. At 7–9 h post-fertilization (hpf), the zebrafish embryos placed in a 12-well plate (15 embryos per group) were treated with EMC (25, 50, and 100 μg/mL). After 1 h, AAPH (15 mM) was added to the medium and the embryos were incubated with AAPH until 24 hpf. And then, the embryos were incubated with the fresh media until analysis. At 2 days post-fertilization (dpf), the heart beating rate of zebrafish was determined. And the intracellular ROS generation, cell death, and lipid peroxidation were determined at the 3 dpf according to the protocol described by Kim et al. [32]. The relative fluorescence intensities of whole zebrafish body were determined using Image J software.

### 2.8. Determination of the Effect of EMC on α-MSH-Stimulated Melanogenesis 

B16F10 cells were seeded in a 6-well plate and incubated for 24 h. Cells were treated with EMC (25, 50, and 100 μg/mL) and stimulated with α-MSH (50 nM). The α-MSH-stimulated cells were harvested after 72 h incubation. Then, the melanin content and the relative intracellular tyrosinase activity of α-MSH-stimulated cells were determined according to the method described by Heo. et al. [33].

### 2.9. Determination of the Effect of EMC on Photodamage Induced by UVB Irradiation

#### 2.9.1. In Vitro in HaCaT Cells

HaCaT cells were seeded and incubated with EMC (25, 50, and 100 μg/mL). EMC-treated HaCaT cells were irradiated with UVB (30 mJ/cm^2^) in PBS solution (1×). The intracellular ROS level and the viability of UVB-irradiated HaCaT cells were investigated with the DCF-DA and the MTT assay, respectively [15,34]. In addition, the apoptosis body formation in UVB-irradiated HaCaT cells was detected with the Hoechst staining assay according to the protocol described by Wang et al. [15].

#### 2.9.2. In Vitro in HDF Cells

HDF cells were seeded and treated with EMC (25, 50, and 100 μg/mL). EMC-treated cells were exposed to UVB (50 mJ/cm^2^). Then, the intracellular ROS level and the viability of UVB-irradiated HDF cells were determined with the DCF-DA and the MTT assays, respectively [28]. In addition, the collagen synthesis level and the expression of MMPs were assessed with ELISA using the cell culture medium [28,35,36].

#### 2.9.3. In Vivo Assay

At 2 dpf, the zebrafish larvae (10 larvae/group) were treated with EMC (25, 50, and 100 μg/mL) for 1 h and exposed to UVB (50 mJ/cm^2^). The UVB-irradiated zebrafish larvae were further incubated for 6 h. The ROS levels, cell death, NO production, and lipid peroxidation were determined according to the methods described by Wang et al. [35].

### 2.10. Statistical Analysis

The experiments were performed in triplicates. Data are expressed as the mean ± standard error (SE), and one-way ANOVA was used to compare the mean values of each treatment in SPSS 17.0. Significant differences between the means were identified with the Tukey’s test.

## 3. Results and Discussion

### 3.1. Chemical Composition

*E. maxima* is the dominant kelp on the west coast of South Africa, and plays an important role in the South African aquaculture and kelp industries [37]. This seaweed is mainly harvested for alginate extraction and abalone feed. *E. maxima* is rich in bioactive compounds such as phlorotannins, steroids, and polysaccharides [38,39,40]. Especially, *E. maxima* has been reported to contain a high amount of polysaccharides [23]. In the present study, we prepared polysaccharides from *E. maxima* through Celluclast-assisted extraction and ethanol precipitation and evaluated the antioxidant, anti-melanogenesis, and UV protective effects of the polysaccharides to explore their potential in cosmetics. 

As Table 1 shows, the moisture, ash, protein, lipid, and carbohydrate content of *E. maxima* were 37 ± 0.17%, 25.52 ± 0.40%, 12.01 ± 0.18%, 1.04 ± 0.08%, and 51.83 ± 0.48%, respectively. These results indicated that *E. maxima* is rich in minerals and carbohydrates, and further confirmed that it contains a high amount of polysaccharides. As shown in Table 2, the yields of the Celluclast-assisted extract of *E. maxima* (EM) and the crude polysaccharides obtained from EM (EMC) were 28.07% and 19.24%, respectively. The protein contents of EM and EMC were 4.33 ± 0.32% and 2.45 ± 0.28%, and the phenolic contents of EM and EMC were 6.23 ± 0.45% and 4.31 ± 0.16%, respectively. In addition, the carbohydrate contents of EM and EMC were 42.37 ± 0.48% and 69.37 ± 0.16%, respectively. The sulfate contents of EM and EMC were 6.29 ± 0.56% and 10.51 ± 0.23%, respectively. Altogether, EM and EMC contain 48.66% and 79.88% sulfated polysaccharides, respectively. These results demonstrate that the protein and phenolic contents were reduced and the carbohydrate and sulfate contents were enriched during ethanol precipitation. EMC contains near 80% sulfated polysaccharides content and could be thought as sulfated polysaccharides.

**Figure 1:**
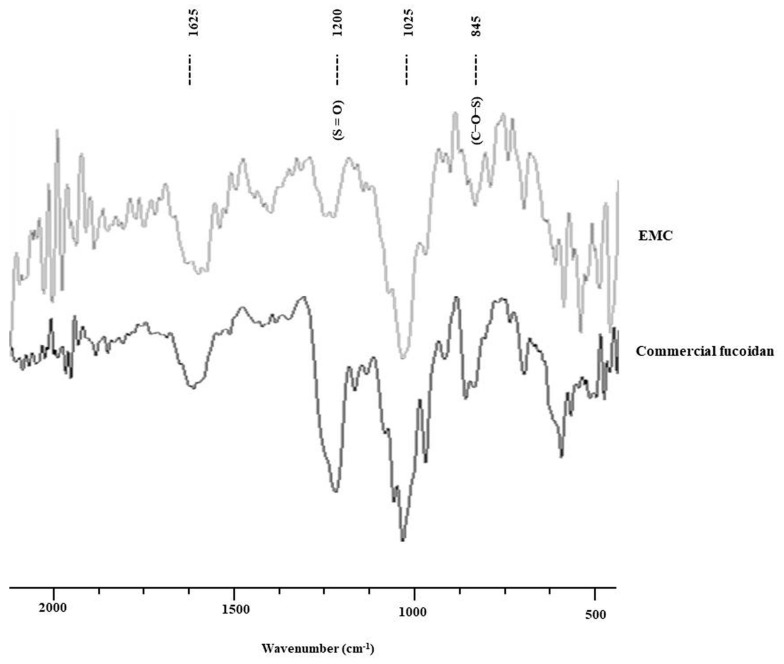
FT-IR spectra of EMC and commercial fucoidan.

EMC was characterized through the FT-IF spectrum and the result was compared to the commercial fucoidan. As Figure 1 shows, the signal at 1625 cm^−1^ was assigned to H-O-H, which indicated the presence of moisture in the sample. The absorption band at 1025 cm^−1^ represented the stretching vibrations of the C-O-C glycosidic band vibration. An intense peak at 1200 cm^−1^ was observed due to the sulfate stretching vibrations (S = O). The bending sulfate vibrations (C-O-S) are represented through the absorptions at 845 cm^−1^. According to these results, we confirmed that EMC is sulfated polysaccharides.

### 3.2. Protective Effect of EMC against AAPH-Stimulated Oxidative Stress 

Oxidative stress is an imbalance between oxidants and antioxidants. It is mainly caused by ROS and could lead to cellular damage [41]. Accumulation of cellular oxidative damage leads to various diseases such as inflammation, cardiovascular diseases, diabetes, obesity, and abnormal aging [13]. Thus, the inhibition of oxidative stress is thought to be a strategy to prevent these diseases as well as against aging. Various studies have reported that the sulfated polysaccharides isolated from seaweeds have antioxidant properties [13,14,26,42]. Wang et al. (2020) isolated the sulfate polysaccharides (CFPS) from the enzymatic digest of *Codium fragile* and evaluated the antioxidant activity of CFPS [14]. The results demonstrated that CFPS remarkably suppressed oxidative stress induced by hydrogen peroxide in both in vitro and in vivo models [14]. Jayawardena et al. (2020) isolated sulfated polysaccharides from *Padina boryana* (PBP) and evaluated the antioxidant activity of PBP in both in vitro and in vivo models. The results indicated that PBP significantly protected Vero cells and zebrafish against oxidative stress induced by hydrogen peroxide [26]. Wang et al. (2019) isolated sulfated polysaccharides from the edible seaweed *Sargassum fulvellum* (SFPS) and investigated the effect of SFPS on AAPH-induced oxidative stress. The results indicated that SFPS effectively suppressed AAPH-induced oxidative stress in vitro in Vero cells and in vivo in zebrafish [42].

**Figure 2:**
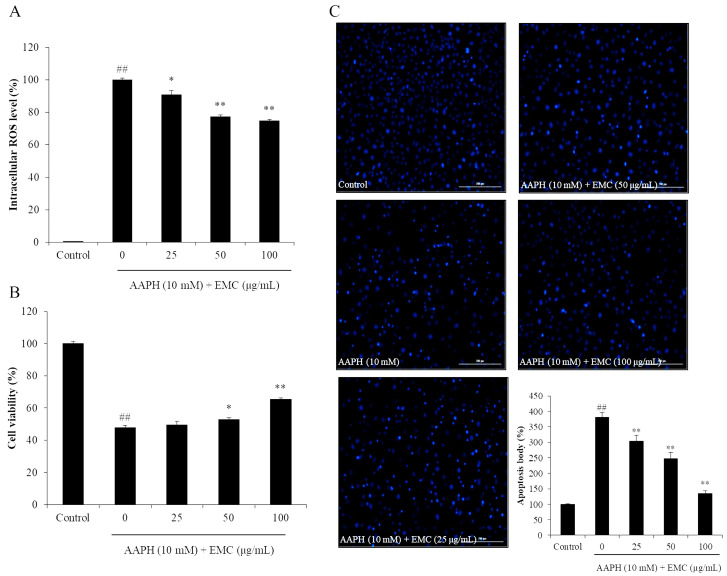
EMC suppresses 2,2-azobis(2-amidinopropane) hydrochloride (AAPH)-induced oxidative damage in vitro in Vero cells. (**A**) Intracellular ROS scavenging effect of EMC in AAPH-stimulated Vero cells; (**B**) protective effect of ECM against AAPH-induced cell death in Vero cells; (**C**) protective effect of EMC against AAPH-induced apoptosis in Vero cells. The intracellular ROS level, cell viability, and apoptotic body formation were evaluated through DCF-DA, MTT, and Hoechst 33342 staining assays, respectively. Apoptosis levels were measured using Image J software. The experiments were conducted in triplicates, and the data are expressed as the mean ± SE. * *p* < 0.05, ** *p* < 0.01 as compared to the AAPH-treated group and ^##^ *p* < 0.01 as compared to the control group.

**Figure 3:**
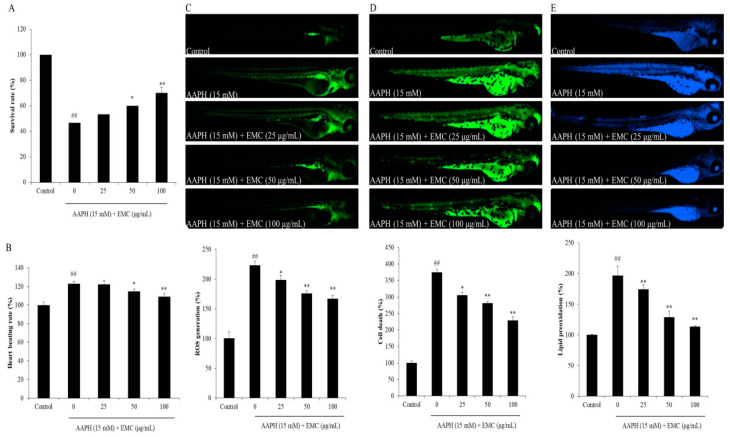
EMC attenuates AAPH-induced oxidative damage in vivo in zebrafish. (**A**) Survival rate of zebrafish; (**B**) heart beating rate of zebrafish; (**C**) intracellular ROS generation of AAPH-stimulated zebrafish; (**D**) cell death of AAPH-stimulated zebrafish; (**E**) lipid peroxidation of AAPH-stimulated zebrafish. The relative fluorescence intensities of whole zebrafish body were determined using Image J software. The experiments were conducted in triplicates, and data are expressed as the mean ± SE. * *p* < 0.05, ** *p* < 0.01 as compared to the AAPH-treated group and ^##^ *p* < 0.01 as compared to the control group.

In the present study, the effect of EMC on AAPH-induced oxidative stress was investigated using in vitro and in vivo models. The in vitro antioxidant activity of EMC was investigated by measuring the intracellular ROS level, viability, and apoptosis body formation of AAPH-stimulated Vero cells. As shown in Figure 2A, AAPH significantly increased intracellular ROS levels in Vero cells. However, the intracellular ROS levels of EMC-treated cells were remarkably and dose-dependently decreased. Additionally, the viability of AAPH-stimulated Vero cells was significantly reduced. However, the viability of AAPH-stimulated Vero cells was effectively improved through the EMC treatment (Figure 2B). In addition, AAPH significantly induced apoptosis in Vero cells. In contrast, the number of apoptotic bodies in EMC-treated cells was remarkably decreased in a dose-dependent manner (Figure 2C). The in vivo antioxidant activity of EMC was investigated using a zebrafish model and the results are summarized in Figure 3. The survival rate of zebrafish exposed to AAPH was significantly reduced but remarkably increased through the EMC treatment in a dose-dependent manner (Figure 3A). In addition, EMC effectively improved the heat beating disorder caused by AAPH (Figure 3B). Further results indicated that EMC remarkably suppressed ROS generation (Figure 3C), cell death (Figure 3D), and lipid peroxidation (Figure 3E) in AAPH-stimulated zebrafish. All effects were dose-dependent. These results indicated that EMC possesses potent in vitro and in vivo antioxidant activities, and suggest the anti-aging potential of EMC.

### 3.3. Anti-Melanogenesis Effect of EMC 

Melanin plays an important role in UV-induced photodamage of the skin. It is also the key pigment that contributes to the color of skin, eyes, and hair in humans [27]. However, overproduction of melanin could cause pigment disorders such as freckles and moles [43,44]. Melanogenesis is a physiological process that involves many melanocyte-related enzymes. Tyrosinase is the rate-limiting enzyme involves melanin biosynthesis. Thus, a compound that effective inhibits the activity or the amount of tyrosinase may have the potential in anti-melanogenesis.

**Figure 4:**
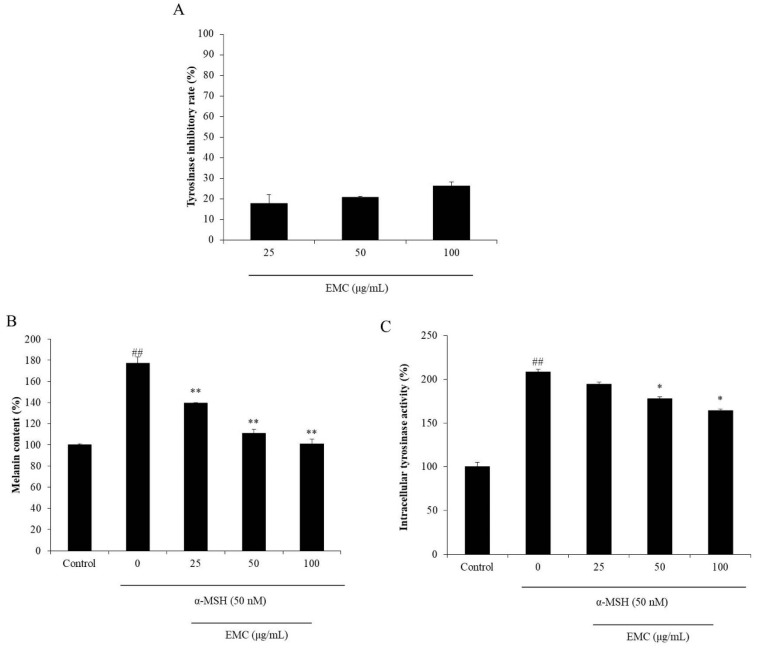
EMC inhibits mushroom tyrosinase and melanogenesis in α-MSH-stimulated B16F10 cells. (**A**) Inhibitory effect of EMC on mushroom tyrosinase; (**B**) inhibitory effect of EMC on α-MSH-stimulated melanin synthesis in B16F10 cells; (**C**) relative intracellular tyrosinase activity of α-MSH-stimulated B16F10 cells. The experiments were conducted in triplicates, and data are expressed as the mean ± SE. * *p* < 0.05, ** *p* < 0.01 as compared to the α-MSH -treated group and ^##^ *p* < 0.01 as compared to the control group.

In the present study, the inhibitory effect of EMC on mushroom tyrosinase was determined. As Figure 4A shows, EMC inhibited 17.87, 20.88, and 26.31% of tyrosinase at the concentration of 25, 50, and 100 μg/mL, respectively. This indicated that EMC could inhibit tyrosinase in a dose-dependent manner and suggest the potential of EMC for anti-melanogenesis. Further results indicated that EMC not only reduced the melanin content, but also suppressed the intracellular tyrosinase activity (Figure 4B,C). Both effects were dose-dependent. These results demonstrate that EMC has an anti-melanogenesis effect and may be a potential ingredient to prepare cosmetics for skin whitening in the cosmetic industry.

### 3.4. Protective Effect of EMC Against UVB-Induced Photodamage

Skin is the largest organ in humans. It covers the surface of the body and is directly exposed to environmental factors such as UV irradiation, chemicals, and environmental pollution. Of these environmental factors, UV irradiation is thought to be the primary factor causing skin damage. Overexposure to UV leads to sunburn, erythema, wrinkling, hyperpigmentation, and skin cancer [34,35]. UVB, a subtype of UV, is thought to cause more stress to the skin than other subtypes of UV. Previous reports suggested that UVB causes both epidermic and dermic damage by stimulating intracellular ROS generation [15,28]. The above data indicated that EMC possesses potent ROS scavenging effect and suggests the photoprotective potential of EMC. In order to investigate the photoprotective effect of EMC, the effect of EMC on UVB-induced damage was evaluated in vitro in HaCaT cells and HDF cells, as well as in vivo in zebrafish.

**Figure 5:**
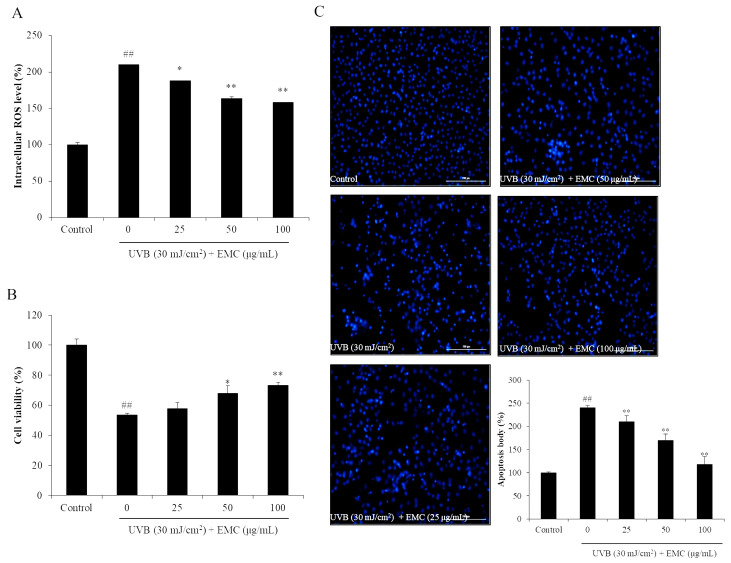
EMC protects HaCaT cells against UVB-induced photodamage. (**A**) Intracellular ROS scavenging effect of EMC in UVB-irradiated HaCaT cells; (**B**) protective effect of ECM against UVB-induced cell death; (**C**) protective effect of EMC against UVB-induced apoptosis. The intracellular ROS level, cell viability, and apoptotic body formation was evaluated by with the DCF-DA, MTT, and Hoechst 33342 staining assays, respectively. Apoptosis levels were measured using Image J software. The experiments were conducted in triplicates, and the data are expressed as the mean ± SE. * *p* < 0.05, ** *p* < 0.01 as compared to the UVB-irradiated group and ^##^ *p* < 0.01 as compared to the control group.

The intracellular ROS level of UVB-irradiated HaCaT cells was significantly increased compared to that in non-irradiated cells (Figure 5A). Intracellular ROS levels were decreased in EMC-treated cells (Figure 5A). In addition, the viability of HaCaT cells was significantly decreased through UVB irradiation while, the viability of cells was remarkably increased through the EMC treatment in a dose-dependent manner (Figure 5B). Furthermore, EMC significantly reduced the apoptotic body formation induced by UVB irradiation (Figure 5C). These results indicated that EMC effectively suppressed epidermic damage caused by UVB irradiation through ROS scavenging.

**Figure 6:**
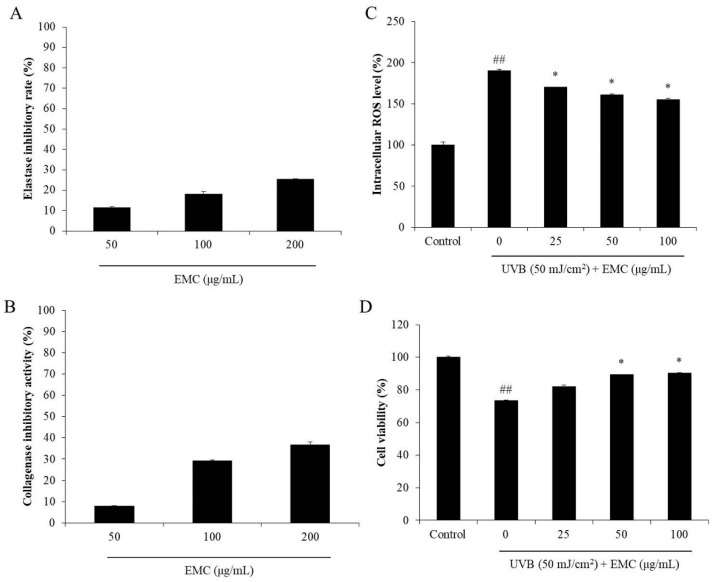
EMC inhibits wrinkle-related enzymes and protects human dermal fibroblasts (HDF) cells against UVB-induced damage. (**A**) Inhibitory effect of EMC on elastase; (**B**) inhibitory effect of EMC on collagenase; (**C**) intracellular ROS scavenging effect of EMC in UVB-irradiated HDF cells; (**D**) protective effect of EMC on UVB-induced cell death in HDF cells. The intracellular ROS level and cell viability of UVB-irradiated HDF cells were evaluated by DCF-DA assay and MTT assay, respectively. The experiments were conducted in triplicates, and data are expressed as the mean ± SE. * *p* < 0.05 as compared to the UVB-irradiated group and ^##^ *p* < 0.01 as compared to the control group.

**Figure 7:**
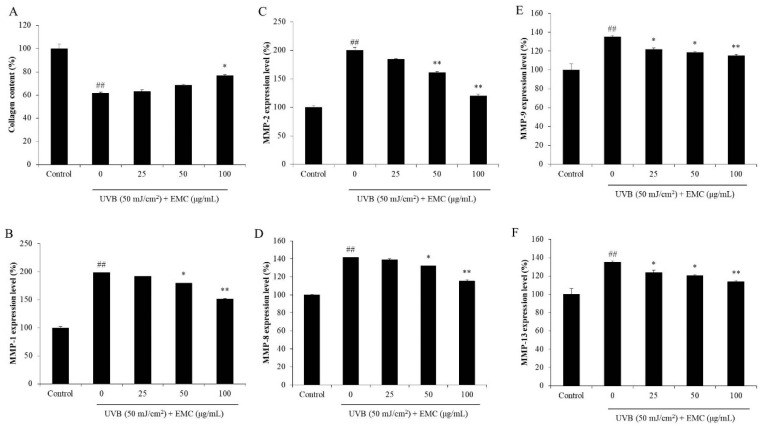
EMC improves collagen content and inhibits the expression of MMPs in UVB-irradiated HDF cells. (**A**) Collagen contents in UVB-irradiated HDF cells; (**B**) MMP-1 expression levels; (**C**) MMP-2 expression levels; (**D**) MMP-8 expression levels; (**E**) MMP-9 expression levels; (**F**) MMP-13 expression levels. The amounts of collagen and MMPs were assessed using the ELISA kits following manufacturer’s instructions. The experiments were conducted in triplicates, and data are expressed as the mean ± SE. * *p* < 0.05, ** *p* < 0.01 as compared to the UVB-irradiated group and *^##^ p* < 0.01 as compared to the control group.

Elastase and collagenase are two proteases that degrade elastin and collagen, which are two important structural and functional proteins in the skin [28]. Degradation of collagen and elastin leads to skin thickness and loss of elasticity, which are the major characteristics of wrinkling in aged skin. Thus, an elastase or collagenase inhibitor may be a potential candidate to decrease skin wrinkling. As shown in Figure 6A,B, EMC inhibited 11.44, 18.05, and 25.51% of elastase and 7.79, 29.25, and 36.79% of collagenase at concentration of 25, 50, and 100 μg/mL, respectively. These results displayed that EMC has the inhibitory effects on elastase and collagenase, and may be a potential anti-wrinkle agent. Therefore, the protective effect of EMC on UVB-induced dermic damage was investigated in further studies. As shown in Figure 6C, EMC significantly decreased the intracellular ROS levels in UVB-irradiated HDF cells. In addition, the viability of UVB-irradiated HDF cells was increased with EMC treatment (Figure 6D). Both effects were dose-dependent. This indicated that EMC protected HDF cells against UVB-induced dermic damage. Furthermore, the collagen synthesis level and MMPs expression levels of UVB-irradiated HDF cells were measured. As shown in Figure 7A, the collagen synthesis level of UVB-irradiated HDF cells was significantly decreased; however, it remarkably increased in EMC-treated cells. In addition, the MMPs expression levels in UVB-irradiated HDF cells were significantly increased especially MMP-1 and MMP-2 (Figure 7B–F). However, the MMPs expression levels in UVB-irradiated HDF cells were effectively and dose-dependently reduced through the EMC treatment (Figure 7B–F). These results demonstrated that EMC protects against UVB-induced degradation of collagen, as well as inhibits the expression of MMPs.

**Figure 8:**
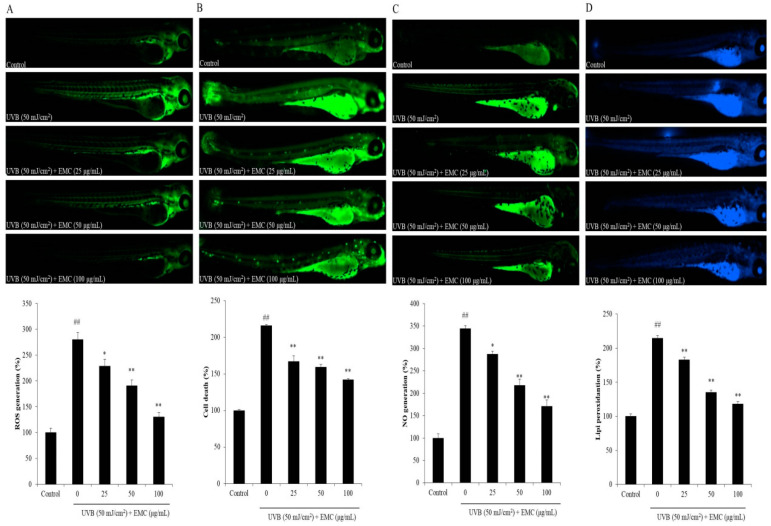
EMC protects zebrafish against UVB-induced damage. (**A**) ROS generation of UVB-irradiated zebrafish; (**B**) cell death; (**C**) NO production; and (**D**) lipid peroxidation. The relative fluorescence intensities of whole zebrafish body were determined using Image J software. The experiments were conducted in triplicates, and data are expressed as the mean ± SE. * *p* < 0.05, ** *p* < 0.01 as compared to the UVB-irradiated group and ^##^ *p* < 0.01 as compared to the control group.

The in vivo photoprotective effect of EMC was investigated in a zebrafish model. As shown in Figure 8, UVB irradiation significantly induced intracellular ROS generation, cell death, NO production, and lipid peroxidation. However, EMC remarkably reduced intracellular ROS level (Figure 8A), decreased the cell death level (Figure 8B), suppressed NO production (Figure 8C), and attenuated lipid peroxidation (Figure 8D) in UVB-irradiated zebrafish. All the effects were dose-dependent. Collectively, these results indicate that EMC has strong photoprotective effect in vitro in both human epidermic and dermic cells, and in vivo in zebrafish model. 

In summary, the above results demonstrated that EMC possesses antioxidant, anti-melanogenesis, and photoprotective effects. It may be a potential candidate for skincare products in the cosmeceutical industry.

## 4. Conclusions

In the present study, the sulfated polysaccharides from *E. maxima* (EMC) were prepared through enzyme-assisted extraction and the cosmeceutical effects of EMC were evaluated using both in vitro and in vivo models. The results suggested that EMC has the potential to suppress oxidative stress, reduce melanogenesis, and inhibit photodamage. The present study suggests that EMC may be used as a cosmetic or a therapeutic agent to prevent or treat skin aging. However, to develop EMC as a therapeutic or cosmetic agent, a clinical study is vital in further research.

## Figures and Tables

**Table 1:** The proximate composition of *Ecklonia maxima.*

Composition	Content (%)
Moisture	4.37 ± 0.17
Ash	25.52 ± 0.40
Protein	12.01 ± 0.18
Lipid	1.04 ± 0.08
Carbohydrate	51.83 ± 0.48

The experiments were conducted in triplicates and the data are expressed as the mean ± SE.

**Table 2:** Chemical composition of EM and EMC obtained from *E. maxima*.

Sample	EM	EMC
Yield (%)	28.07	19.24
Protein content (%)	4.33 ± 0.32	2.45 ± 0.28
Phenolic content (%)	6.23 ± 0.45	4.31 ± 0.16
Carbohydrate content (%)	42.37 ± 0.48	69.37 ± 0.16
Sulfate content (%)	6.29 ± 0.56	10.51 ± 0.23
Sulfated polysaccharides (%)	48.66	79.88

Sulfated polysaccharides = carbohydrate content + sulfate content; EM: Celluclast-assisted extract of *E. maxima*; EMC: the crude polysaccharides obtained from EM. The experiments were conducted in triplicates and the data are expressed as the mean ± SE.
